# Targeting of endothelial cells in brain tumours

**DOI:** 10.1002/ctm2.1433

**Published:** 2023-10-13

**Authors:** Wenzhe Duan, Shengkai Xia, Mengyi Tang, Manqing Lin, Wenwen Liu, Qi Wang

**Affiliations:** ^1^ Department of Respiratory Medicine The Second Hospital Dalian Medical University Dalian China; ^2^ Cancer Translational Medicine Research Center The Second Hospital Dalian Medical University Dalian China

**Keywords:** angiogenesis, brain metastasis, brain tumours, endothelial cells, glioblastoma

## Abstract

**Background:**

Aggressive brain tumours, whether primary gliomas or secondary metastases, are characterised by hypervascularisation and are fatal. Recent research has emphasised the crucial involvement of endothelial cells (ECs) in all brain tumour genesis and development events, with various patterns and underlying mechanisms identified.

**Main body:**

Here, we highlight recent advances in knowledge about the contributions of ECs to brain tumour development, providing a comprehensive summary including descriptions of interactions between ECs and tumour cells, the heterogeneity of ECs and new models for research on ECs in brain malignancies. We also discuss prospects for EC targeting in novel therapeutic approaches.

**Conclusion:**

Interventions targeting ECs, as an adjunct to other therapies (e.g. immunotherapies, molecular‐targeted therapies), have shown promising clinical efficacy due to the high degree of vascularisation in brain tumours. Developing precise strategies to target tumour‐associated vessels based on the heterogeneity of ECs is expected to improve anti‐vascular efficacy.

## Graphical abstract

1. Interactions between endothelial cells (ECs) and tumour cells are involved in most key events during brain tumour development.

2. EC heterogeneity and new research models in brain tumours have received increasing attention.

3. Given the high degree of vascularisation in brain tumours, interventions targeting ECs are of particular importance.

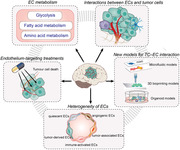

## 1 INTRODUCTION

Aggressive brain tumours, mainly primary gliomas and secondary metastases of various extracranial tumours, are fatal and leave patients with very short survival periods. Glioblastoma (GBM) is the most frequent and aggressive primary brain tumour in adults, and brain metastasis remains a destructive complication of systemic malignancies, which affects about 20% of all cancer patients.^1^, ^2^ Unlike any other tissue or microenvironment, the brain tissue landscape is highly vascularised, providing ubiquitous opportunities for tumour cells to communicate with endothelial cells (ECs).^3^ Microvessels in the tumour microenvironment (TME) assist tumour cells in achieving rapid infiltration and vascularisation through the generation of new blood vessels (tumour angiogenesis)^4^ or dependence on existing blood vessels (vascular co‐option),^5^ eventually causing irreversible damage.

**FIGURE 1:**
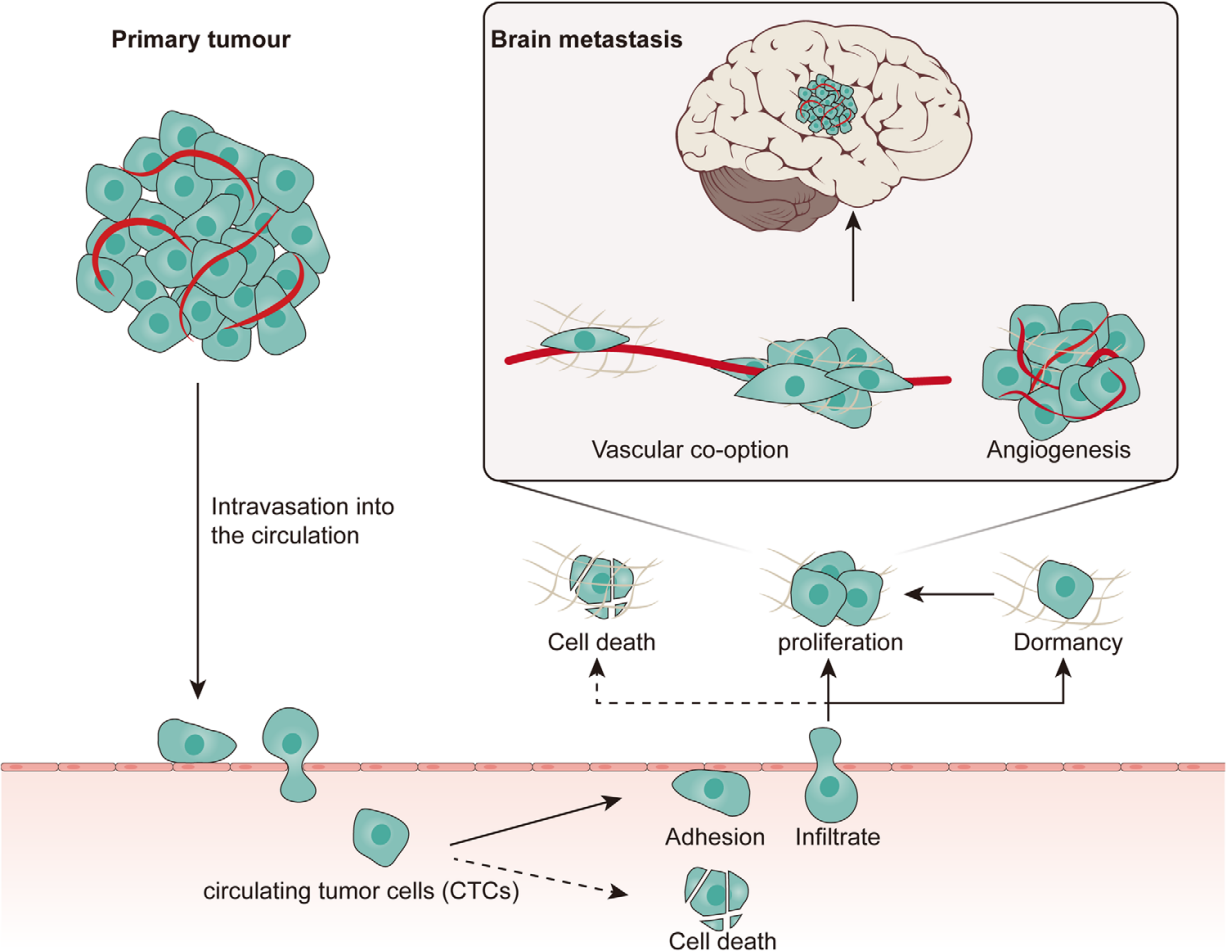
Key events of the brain metastasis process.

ECs play essential parts in all critical events of the brain metastasis process, mainly the following: (1) tumour cells breakthrough from in situ locations into the vasculature (with blood flow to the brain) via intravasation, (2) entry into the blood as circulating tumour cells (CTCs), (3) tumour cell adherence and survival in the vascular niche, (4) breaching of the blood–brain barrier (BBB) and (5) intracranial colonisation and infiltration (Figure 1). On the one hand, ECs protect the brain microenvironment from external interference by forming the BBB, which requires tumour cells to adhere to and break the BBB as the rate‐limiting step for brain metastasis. On the other hand, ECs help to complete dormant tumour cell activation and facilitate subsequent intracranial growth and spread. With the identification of the important role of ECs in brain tumour formation and progression, we review the involvement of ECs and their interactions with tumour cells in all stages of the process and associated research topics that are receiving increasing attention, such as EC metabolism, heterogeneity, new research models and EC‐targeted therapies, to promote a comprehensive and up‐to‐date understanding of the role of endothelium in brain tumours, and provide promising directions for related research.

## 2 EC–TUMOUR CELL INTERACTION

**FIGURE 2:**
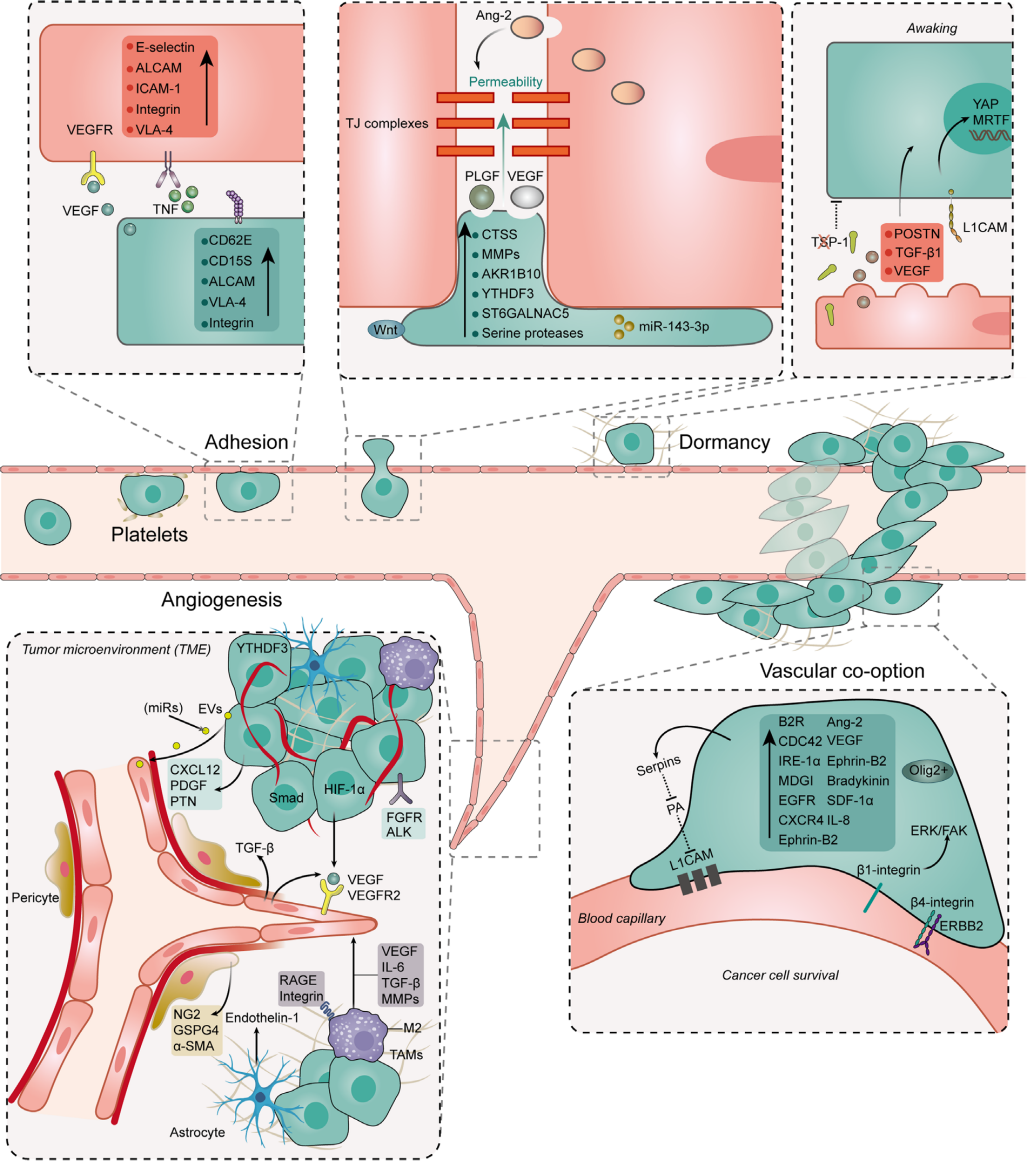
Interactions between endothelial cells and tumour cells in brain tumours. VEGF, vascular endothelial growth factor; VEGFR, vascular endothelial growth factor receptor; ALCAM, activated leukocyte cell adhesion molecule; ICAM‐1, intercellular cell adhesion molecule‐1; VLA‐4, very late antigen‐4; CD, cluster of differentiation; Ang‐2, angiopoietin‐2; tight junctions:tight junction; PLGF, placental growth factor; Wnt, wingless/integrated; CTSS, cathepsin S; MMPs, matrix metalloproteinases; YTHDF3, YT521‐B homology N6‐methyladenosine RNA binding protein F3, ST6GALNAC5, 2,6‐sialic acid transferase; AKR1B10, aldehyde ketone reductase 1B10; miR, microRNA; YAP, Yes‐associated protein; MRTF, myocardin‐related transcription factor; TSP‐1, thrombospondin‐1; L1CAM, cell adhesion molecule L1; TGF, tumour growth factor; POSTN, periostin; TME, tumour microenvironment; EVs, extracellular vesicles; CXCL12, chemokine C‐X‐C motif ligand 12; PDGF, platelet‐derived growth factor; PTN, Pleiotrophin; HIF‐1, hypoxia‐inducible factor‐1; FGFR, FGF receptor; ALK, anaplastic lymphoma kinase; TGF, tumour growth factor; NG‐2, glial antigen 2; GSPG4, chondroitin sulphate proteoglycan 4; SMA, smooth muscle actin; RAGE, receptor for advanced glycation end products; TAMs, tumour‐associated macrophages; IL, interleukin; PA, plasminogen activator; B2R, bradykinin receptor‐2; IRE, inositol‐requiring enzyme; MDGI, mammary‐derived growth inhibitor; EGFR, epidermal growth factor receptor; SDF, stromal cell‐derived factor; CXCR4, CXC receptor‐4; FAK, focal adhesion kinase; ERK, extracellular signal‐related protein kinase; ErbB2, erythroblastic oncogene B2.

A recent study that combined mass spectrometry imaging and single‐cell sequencing techniques to perform cytological analysis of primary and secondary brain tumour samples found that in both primary and metastatic tumours, ECs show strong interactions with cancer cells.^6^ By using multiphoton laser scanning microscopy, Kienast et al.^7^ found that cancer cells that successfully infiltrate the brain usually adhere to the surfaces of capillaries and grow in clusters around these vessels, forming grooves, while those that fail to adhere to the blood vessels are generally eliminated. They visualised the key steps in brain macro‐metastasis formation: tumour cells lodge on microvessels, extravasate through the BBB, spread at perivascular sites in the brain parenchyma and proliferate through angiogenesis or vascular co‐option. Here, we discuss the involvement of ECs in these processes (Figure 2).

### 2.1 Vascular adhesion of metastatic tumour cells

Brain metastasis is a complex cascade of processes in which tumour cells are reprogrammed to enable their metastasis from primary tumours to distant organs and tissues. Although the process is not efficient, some tumour cells responsible for brain metastases formation survive in the circulation and eventually adhere to endothelial niches in the cerebral vasculature, subsequently forming secondary tumours.^8^ Two main mechanisms of CTC vascular arrest have been proposed: (1) physical occlusion of capillaries with diameters smaller than CTCs and (2) ligand expression by CTCs binding to EC surface receptors in capillaries with larger diameters.^9^ Initially, platelets were believed to enhance CTC adhesion to passive vascular endothelium.^10^ However, studies increasingly show that bilateral cell surface receptors and adhesion molecules mediate the adhesion of CTCs to brain ECs. Soto et al.^11^ investigated the spatiotemporal expression of 12 key cell adhesion molecules (CAMs) during the early stages of tumour seeding using two brain metastasis models. They determined that the expression of E‐selectin, vascular CAM‐1, activated leukocyte cell adhesion molecule (ALCAM), intercellular CAM‐1, very late antigen‐4 (VLA‐4) and β4 integrin in ECs is significantly increased in the early stage of tumour seeding, and that the expression of the corresponding natural ligands is up‐regulated on metastatic tumour cells. Two of these ligands (ALCAM and VLA‐4) are particularly highly expressed in tumour cells, and the neutralisation of either ligand with antibodies has been found to significantly reduce tumour seeding in the brain.^11^, ^12^ The essential roles of cluster of differentiation 15s and 62 antigen‐like family member E in non‐small cell lung cancer (NSCLC)‐derived brain metastasis in mediating tumour cell adhesion to brain endothelium have recently been identified.^13^ This interaction is localised to metastatic lung cancer cell–brain endothelium adhesion sites, and the cluster of differentiation 15s immunoblockade significantly reduces such adhesion. β1 integrin on metastatic cells plays a key role in governing interactions with different components of the basal lamina of brain capillaries. β1 integrin on aggressive tumour cells is important in mediating the interaction with matrix components of the cerebral vasculature. Interference on the β1 integrin in breast cancer and melanoma prevents adhesion to the vascular basement membrane and attenuates the formation and growth of metastases in vivo.^14^ β4 integrin enhances the erythroblastic oncogene B2 (ErbB2)‐dependent vascular endothelial growth factor (VEGF) expression by tumour cells, promoting their adhesion to microvascular endothelium. Inhibition of ErbB2 on tumour cells or VEGF receptor 2 on ECs prevents the adhesion of breast tumour cells to the endothelium.^15^ Inflammatory environment–derived tumour necrosis factor‐α promotes tumour cell adhesion by inducing the increased expression of adhesion molecules in ECs and the corresponding ligands in tumour cells.^16^

As vascular adhesion is the first step in initiating metastasis in distant target organs, the leading cause of death in many cases of primary malignancy, its inhibition is a good strategy. Based on this principle, a novel chemoprevention paradigm for cancer metastasis has recently been proposed.^17^ It differs from conventional strategies in that it emphasises the use of drugs to block the vascular adhesion of CTCs, thereby preventing the initiation of the lethal metastatic cascade.

### 2.2 The BBB and blood–tumour barrier

The BBB comprises tight junctions among the endothelium, basement membrane, peripheral feet of astrocytes and pericytes. The structure and function of BBB are highly specific. Although the features of the BBB persist during primary and metastatic brain tumour development, the barrier exhibits functional and structural heterogeneity during these processes, manifesting as increased and uneven permeability, and is regarded as blood–tumour barrier.^18^ Neurovascular unit integrity and vascular permeability are disrupted and impaired in the blood–tumour barrier, with the displacement of astrocytes and pericytes, neurovascular uncoupling and alterations of pericyte populations, tight junctions between ECs and intra‐ and intercellular molecular transport. Significant differences in permeability characteristics and molecular efflux behaviour between the BBB and blood–tumour barrier have led to new strategies for CNS drug delivery.^19^ For ECs, the existence of tight junctions and the absence of extensive cellular phagocytosis and window pore structures, which exist in the peripheral capillary endothelium and allow the exchange of molecular substances, are responsible for protecting the brain from exogenous cell invasion. Studies increasingly show that tight junction disruption is important for metastatic tumour cells’ extravasation through the BBB. Metastatic cells destroy the tight junctions complex by producing and releasing proteolytic enzymes (e.g., serine proteases,^20^ cathepsin S,^21^ and matrix metalloproteinases [MMPs]).^22^ Specifically, YT521‐B homology N6‐methyladenosine RNA binding protein F3 (YTHDF3) and the 2,6‐sialic acid transferase (ST6GALNAC5) were recently proven to promote tumour cell BBB extravasation in breast cancer‐derived brain metastasis,^23^, ^24^ and aldehyde ketone reductase 1B10 (AKR1B10),^25^ placental growth factor^26^ and the non‐coding microRNA (miR)‐143‐3p^27^ were determined to facilitate lung cancer‐derived brain metastasis. EC alterations can also lead to impaired BBB integrity. Avraham et al.^28^ demonstrated that angiopoietin‐2 (Ang‐2) expression was elevated in ECs, impairing tight junction structures and increased BBB permeability. Although cells from primary brain tumours are not expected to penetrate the BBB, this barrier still exhibits significant heterogeneity in these cases. With gliomas, the destruction of the BBB is heterogeneous, depending on the progression stage, and occurs mainly in the tumour core. It is caused by the activated angiogenesis induced by elevated VEGF in hypoxic regions where immature and permeable vessels accumulate and is associated with greater malignancy.^29^ In the four histological/molecular subtypes of medulloblastoma, ECs also show varying degrees of fenestration, affecting drug penetration and treatment outcomes. In the wingless/integrated (Wnt) subtype, the activated Wnt/β‐catenin pathway causes the down‐regulation of claudin5 and SLC2A1, resulting in numerous fenestrations in the vasculature.^30^ In contrast, the sonic hedgehog subtype is distinguished by excessive activation of the sonic hedgehog pathway and BBB intactness, thought to be due to the involvement of sonic hedgehog activation in the development and maintenance of the integrity of the cerebral vasculature.^31^ The disruption of the BBB produces a double‐edged effect, leading to tumour development and facilitating the extensive penetration of macromolecular drugs into the tumour region, often leading to better therapeutic outcomes. Thus, different clinical treatments should be adopted for different degrees of tumour heterogeneity to improve patient prognosis.

### 2.3 Awakening of dormant tumour cells

Before micro‐metastasis formation, disseminated tumour cells that have entered the brain enter a period of dormancy to adapt to the new environment. Intravital imaging has revealed that a small percentage of dormant cells does not tend to proliferate over several weeks; these cells remain solitary or in microscopic clusters with strict perivascular localisation, which may provide optimal conditions for survival.^7^ In this ecological niche, some tumour cells (e.g., those of melanoma) exhibit high degrees of viability while others (e.g., those of lung cancer) remain quiescent. ECs are the first cells contacted by disseminated tumour cells, and these tumour cells that fail to make proper contact with the vasculature (including those of lung and breast cancer and melanoma) die.^7^, ^32^ Disseminated tumour cells undergo apparent spreading across the vasculature exclusively through the cell adhesion molecule L1 (L1CAM), which mechanically activates the Yes‐associated protein (YAP) and myocardin‐related transcription factor (MRTF).^33^ VEGF‐A drives the adaptation of disseminated tumour cells to different vascular interactions,^7^ highlighting their functional dependence on the vasculature. Thrombospondin‐1 (TSP‐1) is identified as an ECs‐derived tumour suppressor. Of importance, TSP‐1 expression is attenuated in the vicinity of the neovasculature, which indicates that tumours may evade growth inhibition in this niche. The time‐lapse analysis further revealed that tumour growth was accelerated significantly around neovascular tips, where tumour‐promoting factors such as active tumour growth factor (TGF)‐β1 and periostin are abundant, indicating that the vascular homeostasis is critical for the preservation of tumour dormancy.^34^

Dormant tumour cells are generally inert and insensitive to external stimuli, including those produced by various treatments, and thus, their presence is a risk for tumour recurrence or metastasis. The generation of unfavourable environmental factors to cause the demise of disseminated tumour cells may thus be another new thought for preventing brain metastasis in the future. Microvascular homeostasis is essential to maintain the dormant ecological niche of disseminated tumour cells, and a systematic understanding of the vascular interactions between disseminated tumour cells and their microenvironment will contribute to the early elimination of tumour metastasis.

### 2.4 Vascular co‐option

Unlike angiogenesis, vascular co‐option means tumour cells achieve infiltration and vascularisation by relying on existing blood vessels. The brain has a minimal connective tissue component that is largely confined to the extracellular matrix (ECM) and external glia surrounding capillaries; the resulting presence of a dense network of blood vessels and basement membrane provides an optimal environment for the vascular co‐option of tumour cells.^5^

#### 2.4.1 Vascular co‐option in GBM

Vascular co‐option has been proven to enable GBM cells to invade the brain, making the treatment of this cancer very challenging. Studies have increasingly revealed that bidirectional remodelling of ECs and GBM cells is important for GBM vessel co‐option. On the one hand, ECs facilitate the vascular infiltration of GBM cells by releasing bradykinin,^35^ stromal cell‐derived factor (SDF)‐1α,^36^ Ang‐2, VEGF,^37^ interleukin (IL)‐8^38^ and ephrin‐B2.^39^ On the other hand, GBM cells with greater vascular infiltration capacity have heterogeneous protein expression profiles. GBM cells with high expression or activation levels of bradykinin receptor‐2 (B2R),^35^ CXC receptor‐4 (CXCR4, the receptor of SDF‐1),^36^, ^40^ epidermal growth factor receptor (EGFR) variant III,^41^ inositol‐requiring enzyme (IRE)‐1α^42^ and ephrin‐B2^39^ have been shown to have high vessel co‐option activity. Notably, Griveau et al.^43^ showed the Olig2‐Wnt7a axis distinctly mediates individual cell vessel co‐option, leading to a more infiltrative phenotype in GBM. They found that astrocyte‐like (Olig2– Wnt7–) GBM cells are predominantly in clusters, whereas OPC‐like cells driven by Olig2–Wnt7a signalling infiltrate blood vessels in the surrounding tissue individually.^43^ Thus, different therapeutic measures need to be taken according to tumour heterogeneity in the future.

#### 2.4.2 Vascular co‐option in brain metastasis

Metastatic cells in the brain are highly dependent on vascular co‐option to maintain tumour progression, particularly in the early stages of colonisation, after the course of extravasation. It is required not only for the survival of metastatic cells after extravasation but also for the growth of disseminated tumour cells upon exit from dormancy.^44^ Kienast et al. indicated that after extravasation, metastatic cells form elongated clusters in close proximity to capillaries, which is known as vascular co‐option. It is thought to be advantageous primarily because it provides disseminated tumour cells with oxygen, nutrients and endothelium‐derived paracrine factors, which are essential for the maintenance and enhancement of the cells’ self‐renewal.^7^ Integrins contribute to this process. Carbonell et al.^14^ demonstrated that β1 integrins induce metastatic breast tumour cells to adhere to the cerebral capillary basal lamina, which in turn causes the activation of focal adhesion kinase and phosphorylation of extracellular signal‐related protein kinase 1/2 to regulate co‐opting cancer cell proliferation. Fan et al.^15^ found that β4 integrins activate the human epidermal growth factor receptor 2 (HER2) expressed on the breast cancer cells to activate the expression of VEGF, thereby mediating tumour cell anchorage to the basal lamina of the brain microvasculature. In addition to integrins, L1CAM is equally important in vascular co‐option in brain metastasis.^32^ Disseminated tumour cells employ L1CAM to spread on capillaries and colonise the brain by mediating β1 integrin‐ and integrin‐linked kinases and thereby activating YAP and MRTF.^33^ Notably, in order to resist the intracranial colonisation of invading tumour cells, brain‐resident astrocytes attempt to block the L1CAM‐mediated vascular co‐option by releasing plasminogen activator (PA), which can cleave and inactivate L1CAM. However, this act of brain self‐defence is thwarted because brain metastatic tumour cells further protect L1CAM from PA effects through secreting anti‐PA serpins (neuroserpin and serpin B2).^45^ Accordingly, drug‐based strategies that target the anti‐PA serpins or L1CAM may disrupt vascular co‐option and prevent subsequent metastasis.^32^

In a word, vascular co‐option is a key step in initiating metastasis and is associated with the alteration of secretory factors, which could be considered potential diagnostic biomarkers to help clinicians dynamically detect tumour dormancy in the future. In addition, drugs that clinically target this process could be developed to avoid large metastasis formation and tumour progression. However, no effective biological strategy has been developed yet. In addition, as the early stages of metastasis are challenging to observe directly in the clinic, developing effective bionic models that mimic vascular co‐option is essential for preclinical studies.

### 2.5 Angiogenesis

Angiogenesis is generally accepted to be governed strictly by a balance of pro‐ and anti‐angiogenic factors. In tumour angiogenesis, this balance is disrupted, and the increased pro‐angiogenic factors lead to the uncontrolled promotion of angiogenesis. These angiogenesis‐related factors can be released by tumour cells and the paraneoplastic microenvironment.

#### 2.5.1 Tumour cell‐derived factors

Angiogenesis is thought to be triggered by hypoxia due to insufficient blood supply caused by rapid tumour growth. Hypoxia induces tumour cells to express hypoxia‐inducible factor‐1 (HIF‐1), which regulates the transcription of many angiogenesis‐related genes, such as VEGF, especially in gliomas. VEGF is the most potent angiogenic factor and the most widely studied. High levels of VEGF promote angiogenesis and tumour cell expansion, whereas low levels lead to reduced tumour vessel growth. VEGF in brain tumours functions in paracrine and autocrine manners by binding with high affinity to VEGF receptor 2/foetal liver kinase receptor 1 and VEGF receptor 1/fms‐like tyrosine kinase‐1.^46^ Auguste et al.^47^ described the role of fibroblast growth factor (FGF)/FGF receptor activity in glioma growth and angiogenesis. TGF‐β‐induced high Smad activity has been shown to be associated strongly with worse GMB prognoses and to promote cell proliferation by inducing platelet‐derived growth factor B (PDGF‐B), which augments glioma angiogenesis through the stimulation of VEGF in tumour ECs.^48^ The expression of the peptide C‐X‐C motif chemokine ligand 12 and its cognate receptor induces and promotes angiogenesis in brain tumours.^49^ Pleiotrophin, a small heparin‐binding cytokine, is overexpressed in high‐grade human gliomas and promotes vascular abnormalisation in correlation with poor patient survival.^50^ Tumour cell‐derived extracellular vesicles and miRs also facilitate brain tumour angiogenesis. Extracellular vesicles mediate intercellular communication by trafficking bioactive molecules (e.g., the secretory factors discussed above and miRs) into the surrounding milieu, thereby promoting glioma angiogenesis.^51^ For example, Lucero et al.^52^ identified eight miRs that may promote angiogenesis in GBM‐derived exosomes, including miR‐9‐5p, which has been proven to maintain angiogenesis levels by regulating the expression of the downstream target genes, sex‐determining region Y‐box 7 and regulator of G‐protein signalling 5. Most current studies of angiogenesis focus on primary brain tumours, especially gliomas; research on the angiogenesis of secondary brain metastasis remains limited. A recent study demonstrated that the N6‐methyladenosine reader YTHDF3 enhances the angiogenesis of breast cancer brain metastasis.^23^ However, the mechanisms underlying the angiogenesis of other metastases need to be explored further.

#### 2.5.2 TME‐derived factors

Tumour cells trigger systemic alterations in the TME to create sites conducive to tumour cell seeding, survival and growth. Multiple studies have shown that various cellular components in the brain TME play important roles in angiogenesis. Astrocytes are the most numerous cells in the brain microenvironment that are responsible for the maintenance of BBB. Astrocytes are activated to tumour‐associated astrocytes in GBM,^53^ while STAT3 labels the reactive astrocyte subpopulations that interact with the metastatic tumour cells and contribute to brain metastasis.^54^ These reactive astrocytes are profiled and shown with the increased expression of pro‐angiogenic factors (VEGF, insulin, albumin and endothelin‐1),^55^, ^56^ indicating a promotive role in angiogenesis. Pericytes, which are important in the modulation of vasoconstriction and vessel permeability, also support tumour angiogenesis. They promote vascular maturation by expressing glial antigen 2, chondroitin sulphate proteoglycan 4 and α‐smooth muscle actin.^57^ Brain malignancies have been found to promote the recruitment of immune cells such as peripheral macrophages (tumour‐associated macrophages [TAMs]) and resident microglia, which usually exhibit an M2 immunosuppressive phenotype. Both cell types secrete angiogenesis‐related factors, such as VEGF, IL‐6 and TGF‐β.^58^, ^59^ TAMs also promote the secretion of MMPs, which are essential for ECM degradation and angiogenesis.^59^ In addition, macrophages in the TME express the receptor for advanced glycosylation end products, which promotes angiogenesis by up‐regulating the pro‐angiogenic factors. The αvβ3 integrin, which is overexpressed by M2‐type macrophages in the glioma microenvironment, has been found to be a pivotal contributor to EC‐macrophage interactions that regulate inflammation‐driven angiogenesis.^60^ A recent single‐cell sequencing‐based study showed a neutrophil cluster characterised by reactive oxygen species‐production in the lung cancer brain metastasis microenvironment. These neutrophils exhibit activated HIF‐1 signalling pathways by expressing HIF1A, lactate dehydrogenase A, VEGF‐A, which are significant pro‐angiogenic factors.^61^ Taken together, angiogenesis is the result of the interaction of multiple TME components. Thus, targeting the TME may provide new ideas for the anti‐angiogenic approaches.

### 2.6 Vasculogenic mimicry

Vasculogenic mimicry is the behaviour of the tumour attempting to form vascular‐like structures to supply nutrients and oxygen to itself, which is not dependent on EC‐based angiogenesis.^62^ Vasculogenic mimicry can occur with various cancer types and is often correlated with a poor outcome. In GBM, highly plastic tumour‐initiating cells may transdifferentiate into ECs and promote angiogenesis.^63^ Evidence for this behaviour has been provided in recent studies. Researchers are currently using the EC markers to identify the structure of vasculogenic mimicry, which is normally tubular.^64^, ^65^ Gliomas contain a wide range of molecular mediators for vasculogenic mimicry. TAMs have been shown to exert a promotive role in vasculogenic mimicry by activating the cyclooxygenase‐2.^66^ Besides, the PI3K/Akt/mTOR signalling pathway is involved in vasculogenic mimicry induction in gliomas.^67^ Combined transcriptomic and epigenomic analyses suggested that Wnt5a pathway activation drives the GBM tumour cells into ECs of cancer cells to endothelial‐like cells.^68^ However, techniques that can clearly distinguish vasculogenic mimicry from normal ECs remain lacking and warrant exploration in future studies.

## 3 HETEROGENEITY OF ECS IN BRAIN TUMOURS

Recent analysis of brain tumours at the single‐cell level has revealed an unexpectedly high level of phenotypic complexity in the TME, including ECs.

### 3.1 Heterogeneity of ECs in GBM

GBM is characterised by broad intratumoural cell heterogeneity. In a native, immunocompetent mouse model of glioma, two endothelial subpopulations are determined. Tumour‐associated vessels make up the majority of the intratumoural vasculature, while tumour‐derived ECs are rare but also contribute to intratumoural vascularisation. There are molecular differences with extensive and significant cellular heterogeneity within the two EC subpopulations. In tumour‐associated vessels, heterogeneous ECs are divided into five clusters based on molecular expression characteristics. The similarity between these clusters is that canonical Wnt signalling and pathways involved in BBB integrity are significantly expressed, suggesting that targeting Wnt or BBB integrity could be used to indiscriminately inhibit tumour‐associated vessels as a routine strategy. In contrast, some potent factors (e.g., non‐coding RNAs, transcription factors) that regulate gene expressions have been found to be active in a certain cluster. It is possible that these potent factors continue to reinforce the transcription of genes that are involved in angiogenesis, such as VEGFR2, and this heterogeneity is likely to account for the failure of anti‐vascular therapies; thus, it is important to explore the mechanisms by which these potent factors regulate endothelial behaviour. Moreover, another cluster exhibits a marked immunomodulatory heterogeneity characterised by the expression of the chemokine receptor cluster of differentiation 74 and the proinflammatory chemokine CXCL10, suggesting an interaction with the immune population, which may explain the emerging immune phenotype. Tumour‐derived ECs present a much stronger infiltrative and angiogenic profile than tumour‐associated vessels, possibly because these ECs transformed by tumour cells still retain the tumour stemness.^63^ In clinical GBM patient tumour samples, five clusters of ECs at different anatomical sites (tumour core or margin) present different phenotypes, including relatively quiescent, angiogenic, intermediate and immune‐activated phenotypes. Notably, the cluster with relatively quiescent phenotype expresses more complete gene expression profiles of normalised vessels as well as BBB integrity (e.g. kruppel like factor 2, a transcription factor that maintains ECs quiescence), suggesting that the pathological activation of ECs is critical for BBB disruption as well as blood–tumour barrier formation. The genes expressing BBB‐related transporter (e.g., SLC2A1, ABCG2, ABCB1, SLCO1A2 and ATP10A) are down‐regulated, while the genes associated with endothelium fenestration and transcytosis are up‐regulated in pathologically activated ECs cluster, compared with the relatively quiescent ECs cluster,^69^ suggesting the underlying mechanism of intratumour destruction of BBB, which deserves to be explored in future studies. In addition, the relationships between different EC states and different brain tumour behaviours and treatment responses should be clarified, as they may contribute to the poor efficacy of anti‐endothelial therapy with single anti‐VEGF agents.

### 3.2 Heterogeneity of ECs in brain metastasis

A study involving the single‐cell sequencing of normal tissue, early‐stage lung cancer and metastases from 44 patients revealed eight EC subclusters. Consistent with the EC clusters in GBM mentioned above, a distinct set of tumour‐derived ECs is found in early‐stage lung cancer and lung cancer brain metastasis samples. Transcriptional sequencing indicates that VEGF and Notch signalling are strongly activated and regulate the fate decisions and development of ECs. Differential gene enrichment analysis of tumour ECs further confirms that angiogenic functions in brain metastases are significantly enhanced, indicating that brain metastasis induces analog vascular alterations to adapt to widespread angiogenesis, which has not been further elucidated. Among these differential genes, the up‐regulated insulin receptor in the intratumoural vascular is considered a potential target for therapies.^70^ Spatial single‐cell analysis reveals that the distributions of ECs within brain metastases indicate significant heterogeneity. It was found that ECs in the core of metastases are distributed with more subpopulations with impaired tight junction proteins than those in the tumour margins, which is consistent with the heterogeneous pattern of primary brain tumours. Impaired expression of claudin‐5, a member of the BBB tight junction protein complex associated with vascular permeability, is observed in those EC populations that interact closely with tumour cells in the core of the metastases,^70^ and our previous study demonstrated that brain metastasis initiating cells complete BBB extravasation by disrupting tight junctions^25^; these findings indicate the prospect that tumour cells in the core of metastases retain the characteristics of the brain metastasis initiating cell. Moreover, these theories also support that intracranial colonisation of brain metastatic cells by vascular co‐option originates in areas with diminished endothelial junctions. In addition to the core region, fibrosis is identified as a key feature of the brain TME characterised by EC accumulation. The TME area with a high degree of fibrosis is reprogrammed to be more angiogenic and fibrogenic, along with immunosuppression, indicating that EC distribution influences the fibrotic status of brain metastasis.^71^

In summary, heterogeneity in both expression profiles and distribution of ECs has been found in brain tumours. Similarly, tumour‐derived ECs, which show a strong neovascularisation capacity, are found in both primary and metastatic brain tumours, indicating the importance of this EC cluster in the intracranial context for brain tumour trophic growth, and targeting this EC subpopulation is expected to provide new therapeutic options for brain tumours. In addition, ECs with varying degrees of tight junction protein expression show heterogeneous spatial distributions, which reflects the influence of different tumour regions on BBB permeability. In the intratumour core region, a large number of ECs with impaired tight junctions are found. On the one hand, it reflects that the core of the tumour may retain the characteristics of the tumour‐initiating cells to a certain extent. On the other hand, it indicates that the oxygen and PH concentration gradients in the core region of the tumour may affect the BBB. Furthermore, the heterogeneity in the integrity of these ECs reflects the preference for vascular selection during the initial colonisation of the metastatic tumour cell. Therefore, protecting the integrity of the endothelial barrier is of great importance both for inhibiting the primary brain tumour's rapid intracranial infiltration and preventing the colonisation of metastases. The difference is that these EC heterogeneities do not produce exactly the same effects in primary and metastatic tumours. In GBM, more intratumour ECs exist in those patients with longer survival, and perivascular tumour cells have a reduced Ki67: CC3 ratio (a ratio that reflects active tumour proliferation) compared with tumour cells that avoid blood vessels, which might be caused by perivascular infiltration of immune cells. In contrast, Ki67 expression is significantly higher in tumour cells interacting with ECs in brain metastases.^6^ This variance might be caused by the different immune landscapes between primary and metastatic tumours since vessels are often closely accompanied by immune infiltration,^72^ and it is novel to take a view of ECs heterogeneity to reveal the underlying mechanism of the emerging immune phenotypes in TME.

## 4 EC METABOLISM IN BRAIN TUMOURS

**FIGURE 3:**
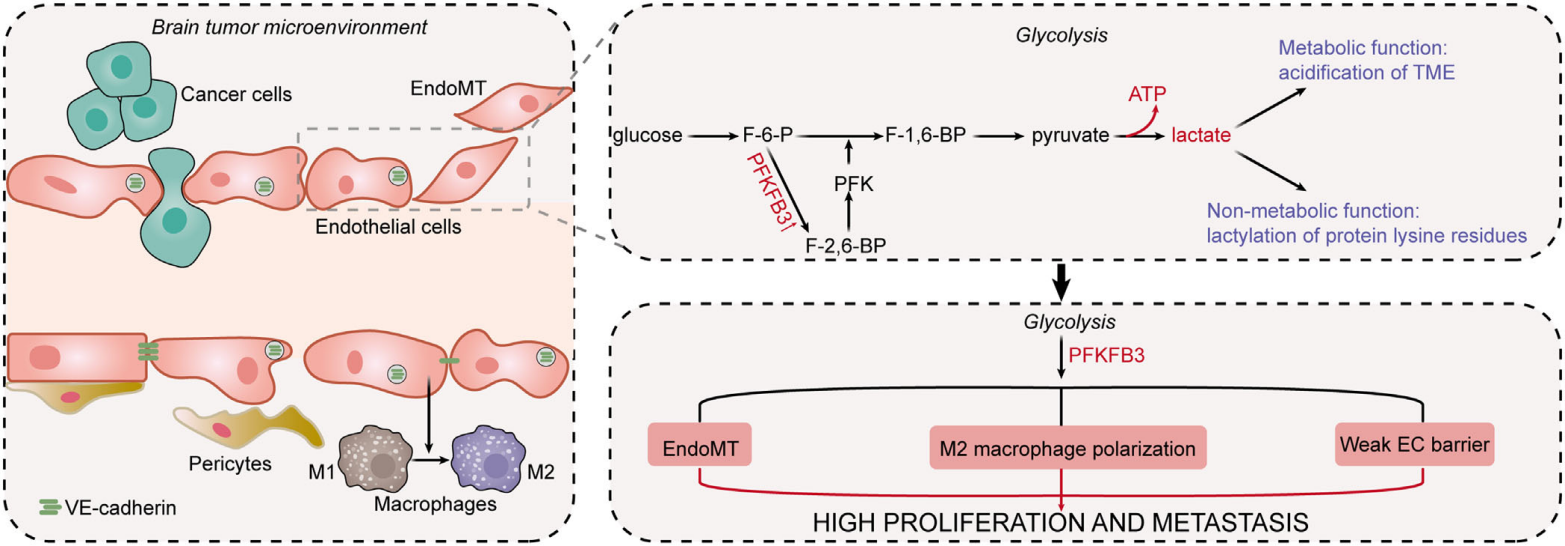
Glycolysis metabolism of endothelial cells in brain tumours. EndoMT, endothelial‐to‐mesenchymal transition; VE‐cadherin, vascular endothelial cadherin; PFKFB3, 6‐phosphofructo‐2‐kinase/fructose‐2,6‐bisphosphatase; ATP, adenosine triphosphate; TME, tumour microenvironment; F‐6‐P, fructose‐6‐phosphate; F‐1,6‐BP, fructose‐1,6‐bisphosphate; F‐2,6‐BP, fructose‐2,6‐bisphosphate; PFK, phosphofructokinase; EC, endothelial cell.

The metabolic reprogramming of the glioma microenvironment has been found to lead to resistance to anti‐angiogenesis drugs, inducing metabolic adaptation to anaerobic glycolysis.^73^ Rather than mitochondrial respiration, ECs rely on glycolysis to produce local adenosine triphosphate (ATP) at the plate and filopods, which increases the migration of apical cells and the proliferation of stalked cells to form blood vessels.^74^ ECs rely on glycolysis to relinquish oxygen to other cells surrounding the blood vessels. Another benefit is the faster kinetics of ATP production by glycolysis, which is important for hypoxic tissues to generate new blood vessels as quickly as possible to escape death. 6‐Phosphofructo‐2‐kinase/fructose‐2,6‐bisphosphatase‐3 (PFKFB3) is the crucial glycolytic activator in ECs. ECs with high PFKFB3 expression exhibit a leading ‘tip’ phenotype, which leads to vascular spout into avascular area and eventual neovessel formation.^75^ PFKFB3 deficiency in ECs and the pharmacological inhibition of PFKFB3 results in vascular deficiency and reduced pathological angiogenesis.^76^ PFKFB3‐driven glycolysis in ECs has been shown to mediate the endothelial‐to‐mesenchymal transition (EndoMT), which is a feature of fibrosis,^77^ as well as the polarisation of M2 macrophages,^78^ suggesting a contribution of ECs in creating a fibrogenic and immunosuppressive TME. In mechanism, PFKFB3‐driven glycolysis mediates the endocytosis of VE‐cadherin (a member of the tight junction protein complex) and makes the pericytes more active, leading to the compromised barrier function of blood vessels.^79^ In addition, lactic acid, as the end product of aerobic glycolysis, has been noted to promote tumour progression. As a metabolite, it causes the acidification of TME and changes in the immune landscape, thereby mediating tumour development and drug resistance.^80^ Recently, the non‐metabolic function of lactate has been emphasised, as it is found to serve as a substrate to induce the lactylation of protein lysine residues (Kla), thus exerting an epigenetic regulatory role and promoting tumour malignant behaviours.^81^ It is reasonable to speculate that lactate production also affects the cell biology and behaviour of ECs by regulating the expression of related genes, which should be explored in future studies. It has been demonstrated that the dual inhibition of VEGF and PFKFB3 enhances therapeutic effects against GBM,^82^ suggesting that targeting glycolysis of ECs might be an effective adjuvant therapy for brain tumours (Figure 3). However, other metabolic pathways (e.g., lipid, amino acid metabolism) of ECs in brain tumours have not been thoroughly investigated. As the brain microenvironment has been shown to be characterised by relatively low lipid levels and serine deficiency, it is likely that tumour‐associated ECs, together with tumour cells,^83^, ^84^, ^85^ undergo reprogramming of lipid and amino acid metabolism to adapt to the microenvironment, which should be elucidated in future studies.

## 5 NEW MODELS FOR THE EXAMINATION OF TUMOUR CELL–EC INTERACTION

In traditional in vitro models, incubation (Transwell) chambers separated by substrate‐coated filters are used to study cellular interactions. However, this approach has limited functional capacity and is far from the in vivo pathological process. In vivo animal models also face ethical limitations. Hence, reliable bionic experimental models that reproduce the complex brain TME, especially vascular structures, including the BBB, are urgently needed. Many studies have recently attempted to construct better in vitro models to simulate tumour cell–EC interactions.

### 5.1 Microfluidic models

**FIGURE 4:**
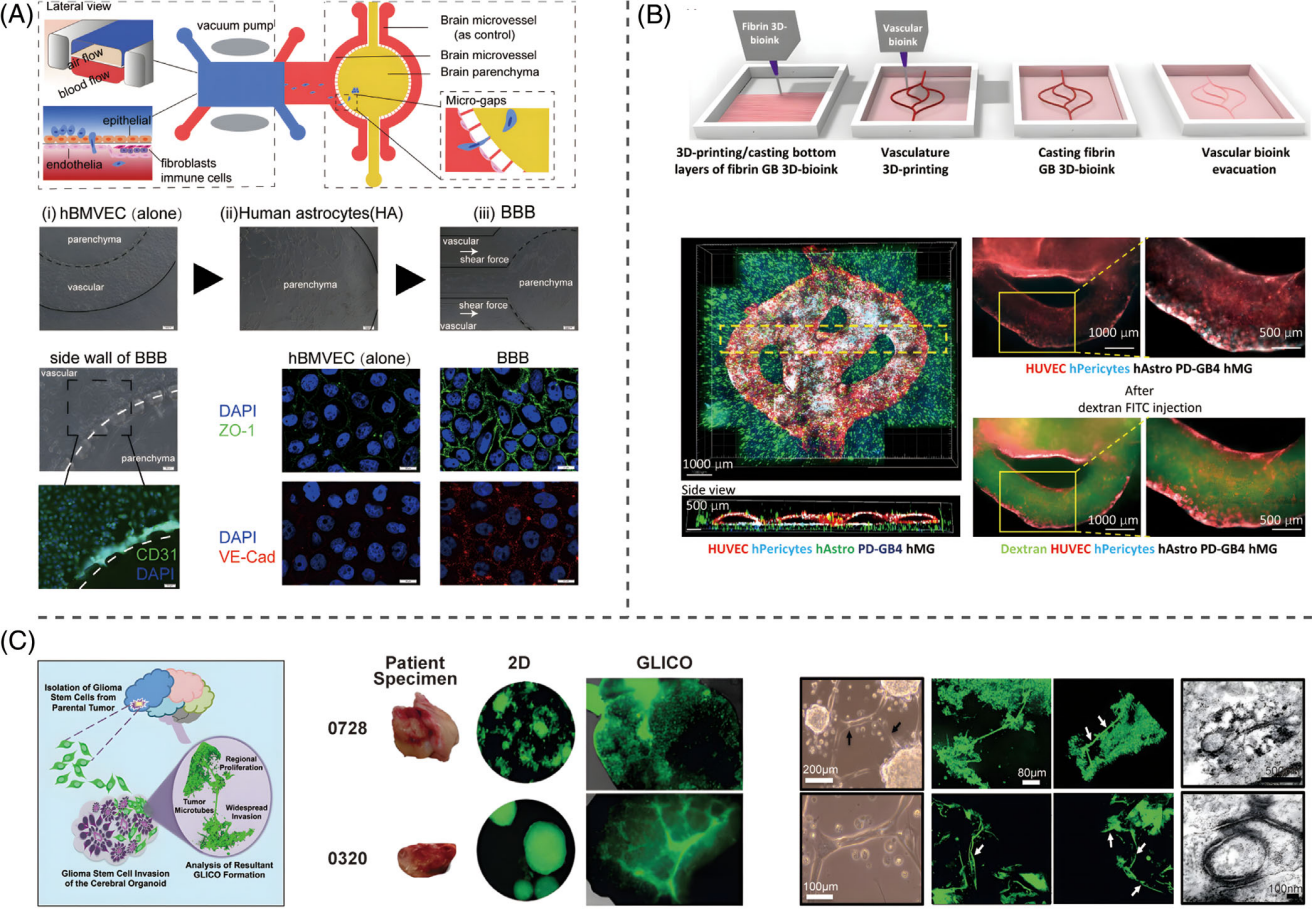
Representative examples of models studying brain tumours. (A) A microfluidic chip for the study of brain metastasis derived from NSCLC is composed of an upstream bionic 'lung' and a downstream bionic 'brain' with a functional ‘BBB’. Reprinted with permission from Ref. 25. (B) A 3D‐printed GBM model that forms a perfused vascular network and a complex tumour microenvironment. Reprinted with permission from Ref. 92. (C) A cerebral organoid glioma (GLICO) model constructed by introducing the patient‐derived glioma stem cells into the established human embryonic stem cell (hESC)‐derived cerebral organoids. Reprinted with permission from Ref. 94.

Microfluidic systems are powerful tools for studying disease pathology and have enabled the establishment of more in vitro models similar to in vivo models. This technology enables complex cellular interactions in vitro by setting up microchannels to manipulate fluids. The most common microfluidic chip model simulating tumour cell–EC interaction is usually designed with several parallel channels connected by dense and equally spaced micro gaps for molecular dissemination and communication. According to the anatomy of tumour cell–EC interactions, there is a collagen channel located between the 'tumour channel' and the 'vascular channel', which both mimic the basement membrane of blood vessels and provide a three‐dimensional (3D) environment to support the activation, outgrowth and angiogenesis of ECs in response to chemokines released by tumour cells. Park et al.^86^ perfused the isolinderalactone into the channel seeded with tumour cells for the examination of the inhibitory effect of isolinderalactone on human GBM growth and angiogenic activity, and Samiei et al.^87^ tested the induction of cell death and the suppression of invasion of anti‐tumour drugs on the chip. It is worth mentioning that the anti‐tumour effects of drugs presented by the microfluidic chips differ from those observed in the traditional drug testing model where tumour cells are cultured alone, suggesting that the TME which contains microvasculature has an impact on the drug sensitivity of tumour cells. These findings indicate that the microfluidic systems can mimic the TME and serve as a promising platform for drug efficacy assessment. Although numerous attempts have been made to mimic the physiological BBB, few studies have concentrated on the dynamic process of and interactions involved in tumour cell breakthrough of the BBB in brain metastasis. Recently, our team constructed a chip to study brain metastasis derived from NSCLC. The chip is composed of an upstream bionic 'lung' and a downstream bionic 'brain' with a functional 'BBB'. This bionic BBB, which is composed of ECs seeded in vascular channels and co‐cultured with the astrocytes seeded in the adjacent brain parenchymal chamber under continuous fluid shear, shows significantly enhanced barrier functions and intact tight junction structures compared with the ECs single layer in vitro (Figure 4A). The chip enables real‐time visual monitoring of the whole brain metastasis process, from primary tumour growth to BBB breakthrough and entry into the brain parenchyma. We have used this chip to demonstrate that AKR1B10 is promotive for tumour cell extravasation through the BBB.^25^ This evidence suggests that microfluidic technology, with its advantage of manipulability, is an ideal platform for constructing physiological structures, such as blood vessels, that require fluid shear stimulation.

### 5.2 3D bioprinting models

3D bioprinting is an emerging technology in which bioactive materials are used as bioinks to construct 3D structures according to a designed model, and it holds great promise for modelling human diseases.^88^ 3D bioprinting enables the better simulation of the microenvironment and the incorporation of perfusable vascular networks into large tissue structures, thus mimicking bidirectional interactions between tumour cells and ECs.^89^ The introduction of decellularised ECM (dECM), a bio‐ink printing material, plays a key role in studying the interaction between tumour cells and ECs in the brain due to its better cytocompatibility.^90^ A bioprinted GBM model reproduced the structure and biophysiology of native GBM by using patient‐derived tumour cells, vascular ECs and dECM from brain tissue as bioinks printed in concentric rings with partitions to create an oxygen concentration gradient.^91^ This model recapitulates the patient's response to the drugs and has great potential for screening effective therapeutic options for GBM. However, the drawbacks of this model are the mere addition of ECs to the system, which fails to form a perfusable vascular channel or network, and the lack of a complex TME. A recent study has attempted to construct perfused vascular networks on 3D printed GBM models by introducing the use of another bioink, Pluronic F127, a temperature‐sensitive reversible and biocompatible synthetic polymer, which is often used as a sacrificial material to form networks or channel structures as its gelation can be reversible by manipulating temperature. Besides, the model forms a complex TME by introducing other brain mesenchymal cells such as astrocytes, microglia and microvascular brain pericytes (Figure 4B). This improved model enables the simulation of tumour heterogeneity, intercellular interactions and spatial tomography. It is more realistic regarding in vivo pathological characteristics and behaviours of tumours, providing a powerful model for drug testing and TME interaction studies in both primary and metastatic brain tumours.^92^

### 5.3 Organoid approaches

Given its complexity, the human brain is difficult to model using other organisms. Intensive research has been conducted to examine the use of in vitro organoid approaches to model human brain development and disease. Bian et al.^93^ established an in vitro 3D neoplastic cerebral organoid model in which brain tumourigenesis was recapitulated via the introduction of oncogenic mutations in cerebral organoids by transposon‐ and CRISPR–CRISPR‐associated protein 9‐mediated mutagenesis. Linkous et al.^94^ established a model system for the retro‐engineering of individual GBMs by introducing the patient‐derived cancer stem cells into the established cerebral organoids, which are derived from the human embryonic stem cell (hESC) (Figure 4C). However, most human brain organoids lack vasculature, meaning that such systems ignore the roles of ECs in disease development. To establish vascularised brain organoids, Mansour et al.^95^ developed a method for transplanting human brain organoids into the brains of mice, and Cakir et al.^96^ engineered hESCs to ectopically express human Ets variant 2, forming a vascular‐like network in human brain organoids. Nevertheless, these improved organoid models have not yet been applied to studying brain diseases, especially brain tumours. Another simple strategy is establishing tumour organoids directly from clinical patient‐derived tumour mass. A tumour organoid is a cluster of tumour‐specific stem cells formed by 3D culturing, which can mimic in vivo tumour characteristics and tumour heterogeneities.^97^ Combining the tumour organoid with recent technologies (such as microfluidic chips or 3D bioprinting), which are capable of simulating complex physicochemical stimulations and TME, has become a preferred option for tumour research.

## 6 ENDOTHELIUM‐TARGETING BRAIN TUMOUR TREATMENTS

### 6.1 Anti‐angiogenic agents

The first‐line conventional treatment for brain metastasis and GBM consists of surgical resection, radiation therapy and chemotherapy. Notably, melanoma‐derived brain metastasis responds favourably to immune checkpoint inhibitors (ICIs) therapy, and long‐term complete responses occur in some cases. Although these treatments do not directly target ECs, they cause complex effects on the vascular system, such as the cerebral vasospasm induced after surgery, the vascular remodelling, leakage and angiogenesis triggered by radiation.^3^ Therefore, endothelium‐targeting therapies should be considered comprehensively, whether administered alone or as an adjunct to these first‐line therapeutic interventions. Bevacizumab is a widely used anti‐angiogenic agent targeting VEGF in clinical practice; however, its therapeutic efficacy is limited. Recent clinical trials have tested strategies combining bevacizumab with other therapies and several other anti‐angiogenic agents to treat these entities (Table 1).

**TABLE 1:** Recent clinical trials for anti‐angiogenic strategies in brain tumours.

Agents	Target	Combination	Phrase	Histology	Outcome	References
Bevacizumab	VEGF	Reirradiation	Phase II	Recurrent glioblastoma	Significant improvement in PFS but no difference in OS	^98^
		Avelumab (anti‐ PD‐L1)	Phase I	Recurrent glioblastoma	PD‐L1 inhibition combined with laser interstitial thermal therapy and using bevacizumab to spare steroids show a good safety profile	^99^
		TVB‐2640 (a FASN inhibitor)	Phase II	High‐grade astrocytoma	A statistically significant improvement in PFS6 over historical bevacizumab monotherapy	^100^
		Vorinostat (a HDAC inhibitor)	Phase II	Recurrent glioblastoma	No improvement in PFS or OS or clinical benefit compared with bevacizumab alone	^101^
		Atezolizumab (A)/carboplatin (C)/paclitaxel (P)	IMpower150	NSCLC with EGFR mutations or metastases in the liver or brain	Although not formally evaluated, an improvement toward delayed time to brain metastasis development was found with ABCP versus BCP	^102^
		Erlotinib	Phase II /III	Untreated, EGFR‐mutated, NSCLC with brain metastasis	An improvement in PFS relative to TKI monotherapy	^103^, ^104^
		Etoposide and cisplatin	phase II	Brain metastasis of breast cancer patients refractory to whole‐brain radiotherapy	Bevacizumab preconditioning followed by etoposide and cisplatin is highly effective	^105^
		Carboplatin	phase II	Brain metastasis of breast cancer	A high rate of durable objective response	^106^
Cediranib	VEGFR	Cilengitide (an integrin inhibitor)	Phase I	Recurrent glioblastoma	Poor survival improvement and response rates	^107^
Ponatinib	VEGFR/FGFR/PDGFR		Phase I	Bevacizumab‐refractory glioblastoma	Minimal activity	^108^
Cabozantinib	VEGFR2/ MET		Phase II	Recurrent or refractory glioblastoma	Clinically active but did not meet the predefined statistical target for success	^109^
		Trastuzumab	Phase II	Breast cancer brain metastasis	Antivascular effects but no sufficient anti‐tumoural activity.	^110^
Trebananib	Ang‐1/2	Bevacizumab	Phase II	Recurrent glioblastoma or gliosarcoma	A detrimental combination with a shorter PFS and no improvement in 6‐month PFS rate or OS.	^111^
Plerixafor	CXCR4	Radiation	Phase I/II	Newly diagnosed glioblastoma	Improved the local control of tumour recurrence	^112^

Abbreviations: Ang, angiopoietin; CXCR4, chemokine (C‐X‐C motif) receptor 4; EGFR, epidermal growth factor receptor; FASN, fatty acid synthase; FGFR, fibroblast growth factor receptor; HDAC, histone deacetylase; MET, mesenchymal–epithelial transition factor; NSCLC, non‐small cell lung cancer; OS, overall survival; PD‐L1, programmed cell death protein ligand‐1; PDGFR, platelet‐derived growth factor receptor; PFS, progression free survival; PFS6, 6 months progression‐free survival; VEGF, vascular endothelial growth factor; TKI, tyrosine kinase inhibitor; VEGFR, vascular endothelial growth factor receptor.

### 6.2 Combined strategies

#### 6.2.1 Combined anti‐angiogenic and immune checkpoint therapy

Anti‐angiogenic therapy with the addition of bevacizumab has not been successful as a first‐line treatment for either GBM or brain metastasis, and new therapeutic strategies are urgently needed. In addition to highly abnormal angiogenesis, GBM is featured by an immunosuppressive microenvironment that hampers matured dendritic cells and the toxicity of T cells. For this reason, immune checkpoint therapy is a novel approach to its treatment. Given the success of ICIs in the treatment of melanoma and lung cancer, especially for melanoma‐derived brain metastasis,^113^ researchers examined tissue sections from GBM patients with bevacizumab treatment and determined that programmed cell death protein‐1 (PD‐1)/PD‐1 ligand signalling was a potential target.^114^ Combined triple therapy with blockade of VEGF (aflibercept), Ang‐1/2 (AMG386) and PD‐1 exhibited a better efficacy and significantly prolonged survival compared with vascular‐targeted therapy alone in a homozygous in situ GBM model. This triple therapy increased the number of cytotoxic T lymphocytes, associated negatively with the numbers of myeloid‐derived suppressors and regulatory T cells in the GBM microenvironment.^115^ A Phase I trial indicated that the addition of bevacizumab to ICIs might be beneficial by reducing cytokine and immune cell expression in recurrent GBM, suggesting that bevacizumab exerts a modulatory effect on immune cells and related cytokines, which can enhance the effect of ICIs.^99^ Gutin et al. demonstrated that patients with GBM tolerate the combination of bevacizumab and hypofractionated stereotactic radiation therapy (hfSRT) well.^116^ Sahebjam et al.^117^ showed that the use of pablizumab, a PD‐1 inhibitor, combined with hfSRT and bevacizumab treatment, is also well tolerated in patients with recurrent GBM or mesenchymal astrocytoma. These findings provide a theoretical basis for the overcoming of tumour immunosuppression and the limitations of current VEGF monotherapy, as VEGF/Ang‐2 and PD‐1 blockade have synergistic effects that enhance anti‐tumour immunity through immune microenvironmental modulation and vascular normalisation.

#### 6.2.2 Combined anti‐angiogenic and molecular targeted therapy

In GBM, the expression of the mesenchymal–epithelial transition factor (MET) has been widely studied and correlates with bevacizumab resistance. However, a phase‐2 multicentre study suggested that combining bevacizumab with the MET inhibitor onartuzumab did not benefit patients with recurrent GBM.^118^ This might be due to other more potent factors mediating bevacizumab resistance. Nearly 50% of patients with EGFR‐mutated lung cancer develop brain metastasis. Fibroblast growth factor receptors (tyrosine kinase inhibitors [TKIs]) targeting EGFR are widely used in clinical treatment. In a recent phase‐3 multicentre study, treatment with bevacizumab plus erlotinib improved progression‐free survival (PFS) relative to erlotinib alone in Chinese patients with brain metastasis of untreated, EGFR‐mutated, advanced NSCLC, indicating the promise of combined anti‐angiogenic and molecular targeted therapy.^104^ A similar assessment was recently performed in South Korea. Although there was no statistical significance, longer PFS tended to be shown in patients treated with bevacizumab plus erlotinib compared with those treated with erlotinib alone.^103^ Although the first‐generation TKIs in combination with anti‐vascular therapy have shown promising results in lung cancer patients with brain metastasis, a recent phase II study performed in advanced EGFR‐mutated lung adenocarcinoma patients who received prior EGFR‐TKI treatment indicated that bevacizumab plus osimertinib, a third‐generation TKI which is effective for brain metastasis from NSCLC, failed to prolong the PFS in patients.^119^ These findings suggest that bevacizumab is unnecessary for adjuvant combinations of all EGFR‐TKIs. HER2 is an important driving factor and therapeutic target in breast cancer. Although there is evidence that combining dual HER2 inhibition with anti‐VEGF therapy is tolerable and active in breast cancer patients,^110^ the results suggested that cabozantinib is insufficient in patients heavily pretreated with trastuzumab. Since cabozantinib remains more application‐limited than bevacizumab, further investigation is warranted to assess the efficacy of bevacizumab plus trastuzumab in treating breast cancer brain metastasis.

## 7 CONCLUSION AND PERSPECTIVES

**FIGURE 5:**
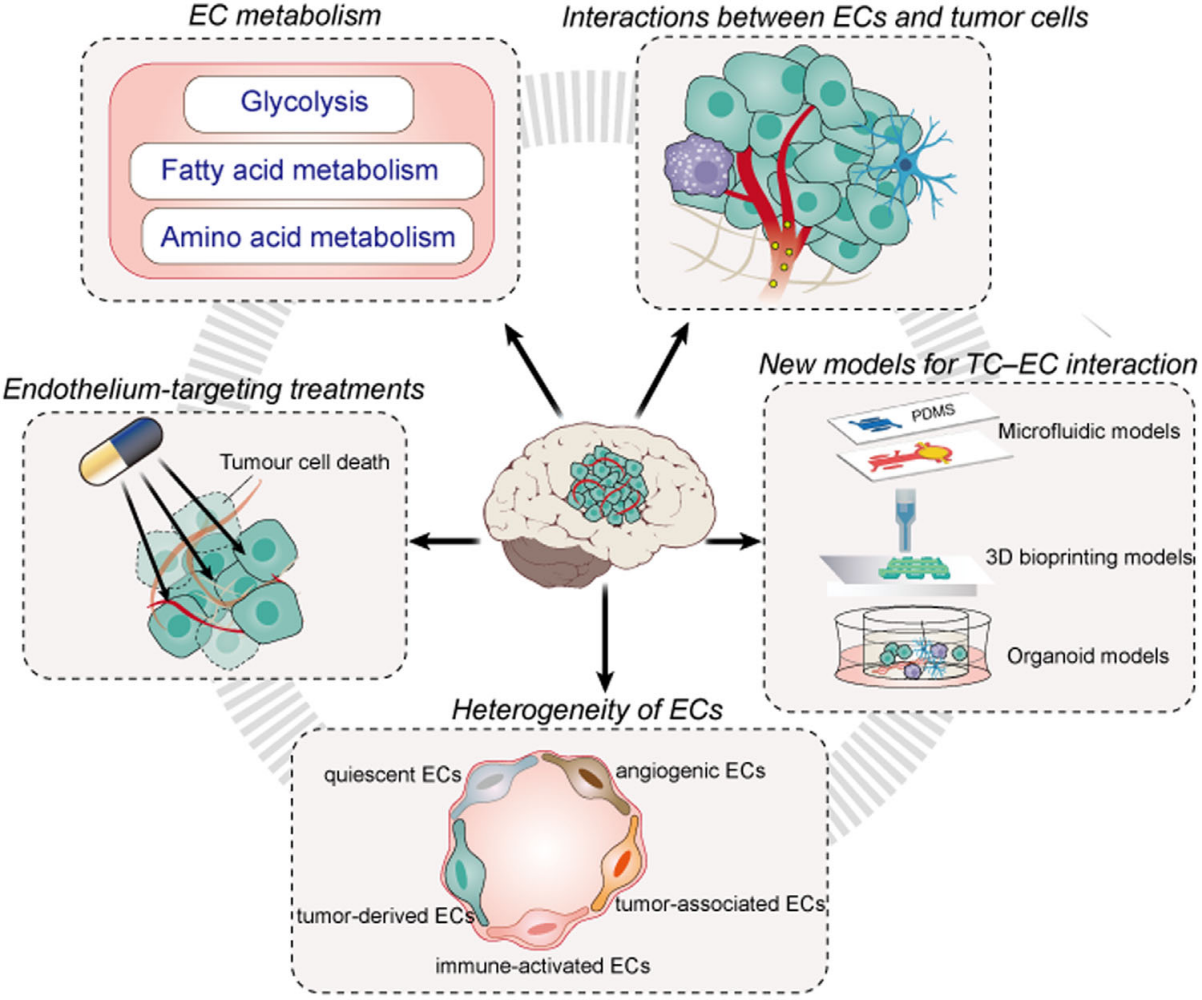
Schematic representation of the content on endothelial cells in brain tumours.

For tumours with a high potential to metastasize to the brain (e.g., lung cancer, breast cancer, melanoma), the majority of cancer patients now die not from the primary disease but from metastasis occurring 1−5 years after the surgical removal of the primary tumours; as current state‐of‐the‐art diagnostic modalities and auxiliary tools enable the precise resection of localised pre‐metastatic tumours. Unfortunately, due to the specificity of the brain microenvironment, both primary brain tumours and secondary brain metastases remain life‐threatening. Future therapeutic approaches for primary and secondary brain malignancies should target not only tumour cells but also other cells in the TME. Given the high degree of vascularisation of brain tumours, interventions targeting ECs are of particular importance. Interactions between ECs and tumour cells are involved in most key events during brain tumour development. However, existing anti‐endothelial treatment strategies are mainly anti‐angiogenic and ignore other key events involving ECs, leading to limitations in therapeutic efficacy. In addition, the heterogeneities of ECs suggest that generalised anti‐endothelial therapy may not lead to specific efficacy and that precise targeting of certain characteristic EC populations may be a better option. Moreover, focusing on the specific metabolic reprogramming that occurs in ECs in brain tumours may also facilitate precision therapies targeting tumour‐associated ECs. With the development of preventive and evidence‐based medicine for secondary brain metastases, clinicians and researchers tend to believe that preventing metastasis is easier than treating it. Therefore, the application of chemopreventive strategies before metastasis occurs provides more possibilities in clinical practice. As the endothelium is a significant driver of brain tumours, we believe that more endothelium‐related research models and preventive therapies will be proposed based on the mechanisms summarised in this review (Figure 5).

## CONFLICT OF INTEREST STATEMENT

The authors have declared that no conflict of interest exists.

## Data Availability

Not applicable.
